# Hair Longevity—Evidence for a Multifactorial Holistic Approach to Managing Hair Aging Changes

**DOI:** 10.3390/jcm14061894

**Published:** 2025-03-11

**Authors:** Gillian E. Westgate, Daniela Grohmann, Manuel Sáez Moya

**Affiliations:** 1 Gill Westgate Consultancy, Stevington, Bedfordshire MK43 7QT, UK; gill@westgateconsultancy.co.uk; 2 Olistic Research Labs SL, 08021 Barcelona, Spain; daniela.grohmann@olisticscience.com

**Keywords:** hair loss, alopecia, metabolism, aging, senescence, hair and scalp

## Abstract

Loss of hair density—hair thinning and balding— is typically referred to as male and female pattern alopecia. Causes include genetic predisposition and links to the impact of dihydrotestosterone on the follicle dermal papilla, which are typically characterized by an increase in the number of vellus follicles. Links to chronological aging are unclear. Proven treatments remain few in number and are still targeting and tested on those experiencing classical pattern hair loss. The way hair changes with aging, especially in women, can be considered as having a much broader scope. Trends in managing changes to hair density, length, and fiber quality with aging now mostly include cocktail approaches—whether topical, injected, or oral—recognizing that solutions are more likely to require a multifactorial strategy. This review examines the evidence for the more holistic approach to addressing unwanted hair loss, which includes nutrition, lifestyle, stress management, and scalp and hair care, as well as co-morbidities with other health concerns. We discuss the strengths and limitations of clinical study design to investigate efficacy using multifactorial holistic approaches. We propose that this strategy will contribute to the emerging concept of hair longevity in which follicle, scalp, and fiber are targeted and that maintaining anagen is the most appropriate route to achieving healthy hair with aging. Finally, we discuss the problem facing patients and consumers regarding the quantity of misinformation and how it influences choosing from a fast-growing market of solutions that bypass a pharmaceutical approach to hair thinning.

## 1. Introduction

**Figure 1:**
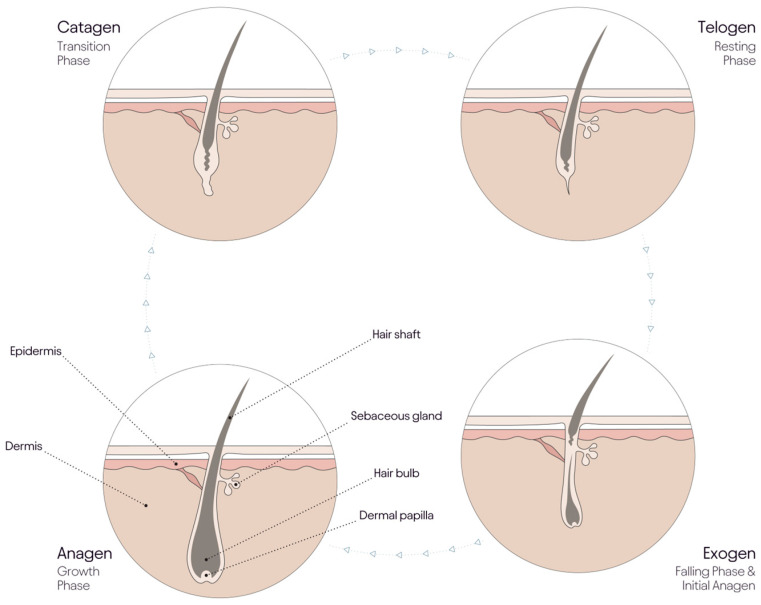
Hair growth cycle. The hair growth cycle consists of the anagen, catagen, telogen, and exogen phases, each contributing to its dynamic progression.

Hair loss—typically referred to as alopecia—occurs in both males and females and affects all racial groups and ages. Male and female hair loss are distinguished by pattern as well as treatment options. In male pattern hair loss, thinning at the vertex and temples is due to androgen-driven follicle miniaturization, whereas, in females, thinning is generally on the crown and midline parting, with retention of the frontal hairline [1,2]. Whilst some follicle miniaturization is seen in female pattern hair loss [3], it is more generalized. Both male and female pattern hair loss respond well to minoxidil [4], which increases anagen density, and males respond well to oral finasteride; however, finasteride is contraindicated in women before menopause, even when formulated for topical use. Hair loss is often reported as a side effect of many physiological events, such as post-partum shedding [5], hormonal and post-menopausal changes [1,6], seasonal hair shedding [7], obesity-related insulin resistance/metabolic syndrome [8], and low ferritin levels in women [9,10,11] and excessively low protein diets [12]. There are around 100,000 hair follicles on the scalp, normally producing approximately 12 km of hair shaft each year during the growth stage of the hair cycle, anagen. Whilst hair shaft formation can continue for several years to produce long scalp hair in over 80% of scalp hair follicles, each follicle periodically shuts down shaft production, enters a regression state (catagen), and the proximal shaft end is transformed into a weakly adherent club hair structure as the hair follicle rests in telogen and are sometimes shed even before the follicle re-enters anagen, Figure 1.

Hair follicles have many highly proliferative cells, a specific type of metabolism [13,14] where aerobic glycolysis and glutaminolysis are used to generate ATP. Follicles are a sensitive barometer in the body to changes that are both internal and external. Sudden stress, a physiological event, or certain drugs can induce an orchestrated shutdown of the hair cycle, which leads to a great increase in hair shedding; this is a most concerning symptom of hair loss [15]. Furthermore, in pattern hair loss, there is a replacement of larger follicles by small, ‘vellus’ follicles, which produce short, fine, unpigmented hair. It is the increase in these vellus follicles that gives rise to bald/thin patches in males and females [16].

Any hair loss and thinning can be traumatic for the person, and great efforts from basic and clinical researchers over decades have been made to identify the causes of the main types of hair loss as well as clinically test new therapies. These include (i) Jak inhibitors which are the latest promising routes to hair regrowth in alopecia areata [17]; (ii) 5-α reductase inhibitors, finasteride & dutasteride for male pattern hair loss, which is strongly linked to a predisposition to androgen sensitivity in hair follicles in the vertex, temporal and frontal scalp regions [18]; (iii) minoxidil, developed for topical use for over 30 years for both male and female pattern hair loss and proposed to increase blood flow to the follicles, but also has a suspected local action on the follicle cells based on its potassium channel agonist action [19,20,21,22]; (iv) physical approaches, the most commonly used of which is low-level light therapy which works through poorly understood mechanisms but is often effective when combined with other treatments [23,24] and (v) a multitude of ‘other’ treatments with variable proof of efficacy. Some scarring types of alopecia, for which less is understood about the etiology and for which even fewer proven therapies exist, form important subsets of alopecia [25,26]. Once hair thinning/balding is diagnosed, the treatment seems to require continuous use, especially for pattern hair loss, and stopping treatment can cause a reversion to the original state of hair thinning or worse.

Hair longevity is a relatively new term that has been used in reference to a more holistic approach to aging-related hair concerns. In most forms of hair loss, whether due to a change in the follicle or to damage and breakage of the hair shaft, the links to chronological aging are unclear. Hair thinning with age, especially in women, is a significant worry, specifically for those not wishing to take a ‘drug’ continuously for life. So, the way hair changes with aging, especially in women, can be considered as having a much broader scope and falls under the relatively new term ‘hair longevity’. Trends in managing changes to hair density, length, and hair fiber quality with aging now mostly include ‘cocktail’ approaches, whether oral, topical, injected, and/or nutritional, recognizing that solutions for healthy hair throughout life are more likely to require a multifactorial strategy which can vary within the person’s life and life stage. 

The hair fiber itself also ages over time. Principal factors in hair fiber aging are exposure to UVR, pollution, chemicals, appliances, and heat. Repairing damage to the hair fiber itself has recently become the focus of technologies called bond-builders [27] that interact with the fiber to improve its resilience and appearance. 

So, hair longevity should be seen as a combination of hair growth (anagen) density, maximum length of each hair fiber, diameter and color, and resilience to day-to-day grooming and product use, and it should be relevant at any age. Hair fibers longer than 12 cm will start to show some signs of damage at the distal ends due to grooming and product use. This is increased with chemical treatments [27,28]. Thus, maintenance of healthy hair is now considered the ideal, and so-called ‘hair longevity’ requires attention to multiple factors that can impact both hair growth and hair fiber. These will be discussed below. 

## 2. Methods

This is a non-systematic review in which the available literature on cellular and biological aging, hair aging, pattern hair loss, and hair fiber aging/damage was analyzed. A literature search was conducted using MEDLINE via PubMed (National Library of Medicine, Bethesda, MD, USA) and Google Scholar (Google LLC, Mountain View, CA, USA) databases from the present day (January 2025) back to the 1950s when early concepts on hair follicle metabolism emerged. Keywords used were hair aging, hair longevity pattern, hair loss, hair nutraceuticals, hair fiber damage, hair cycle, and hair follicle metabolism. Unless there is a direct link to the narrative, data on scarring alopecia and alopecia areata were excluded, as were physical treatments for hair loss, as these have been covered already in this series [29]. Conference abstracts and articles not in English were excluded.

## 3. Results

### 3.1. Aging and the Hair Cycle

**Figure 2:**
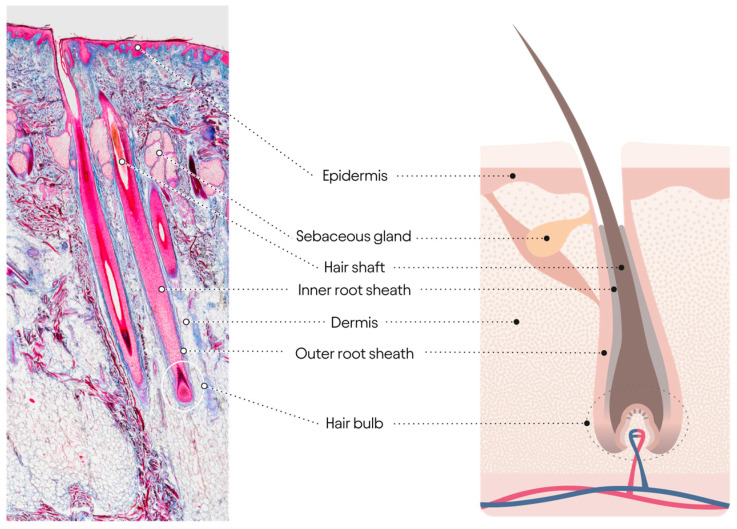
Hair follicle anatomy: a histological representation of the hair follicle is shown on the (**left**), while a diagram representation is displayed on the (**right**).

The hair cycle in mammals describes the stochastic transition between periods of hair fiber formation (anagen) and hair fiber anchorage (telogen), with catagen being the transitional process between anagen and telogen and exogen being the active shedding of the telogen hair fiber. In the human scalp, most follicles are large, set deep in the fatty layer of the scalp skin, and synthesize a substantial diameter hair fiber (Terminal hair 80 μm), which can grow to a great length (+/−100 cm), Figure 2. Thus, scalp hair cycles are long (several years), and transitions between cycles are short (12 weeks). Lengthy anagens mean fewer shedding instances, and the ‘dogma’ is that up to 100 hairs from the approx. 100,000 follicles are shed daily.

**Figure 3:**
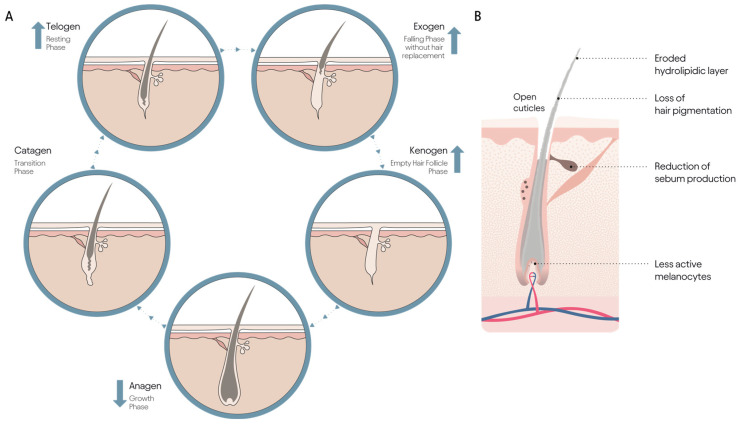
Aged hair growth cycle: displaying the dynamic process of the hair growth cycle, including the anagen, catagen, telogen, exogen, and kenogen phases. (**A**) Emphasis is placed on the changes associated with hair aging, such as a shorter anagen phase (↓), a longer telogen phase (↑), and the increased prominence of the exogen (↑) and kenogen phases (↑). (**B**) Diagram of an aged hair follicle, highlighting the primary alterations caused by the aging process: open cuticles, eroded hydrolipidic layer, loss of hair pigmentation due to less active melanocytes, and reduction of sebum production.

So, what happens with aging and hair loss? Hair aging is often discussed solely in relation to alteration in the dynamics of the follicle hair cycle related to pattern hair loss that gives rise to hair thinning (less density) together with a reduction in the size of the follicle and its re-location nearer the skin surface [16,30,31,32]. Indeed, shortening of the anagen phase, increasing numbers of hair cycles over time, lengthening of the telogen phase, and increases in the numbers of ‘empty’ follicles are all characteristic of pattern hair loss in women, Figure 3A [33,34,35,36]. The higher proportions of telogen and weakly anchored telogen scalp hairs that are shed well before the follicle is triggered back into anagen is the earliest sign of a change in hair cycle dynamics. Hair changes in the very elderly are sometimes referred to as ‘senescent’ alopecia, Figure 3B [37,38]. This is distinct from pattern hair loss and balding with density, color, and diameter of hairs reduced, whilst anagen duration can remain sufficient for shoulder length or longer hair growth without an obvious hair loss pattern. The typical white, wispy, longer hair of elders reflects this type of hair aging, and this has long been proposed to be associated with chronological aging in both men and women [39].

### 3.2. Cellular Aging and Relevance to Follicle Processes

Aging (senescence) is a chronic process that reflects a gradual accumulation of damage to DNA, cell processes, structural components of the extracellular matrix (ECM), tissues, and organs [40,41,42]. Genomic factors such as telomere shortening and hypermethylation disrupt normal cell functions and are among the biomarkers characteristic of aging cells [41]. Chronic aging also results in lower levels of ATP generation in the epidermis due to reductions in ATP synthase subunit [43]. Age-related reduction in epidermal regeneration alters epidermal morphology and thinning of the epidermis, including on the scalp [44]. The hair follicle is unusual in that it can fully regenerate several times in the lifespan of the adult, which may suggest that the longer-term impacts of aging with effects are limited to follicles’ time in anagen. However, we hypothesize that chronological aging does, in fact, impact hair aging by effects on cells, leading to a reduction in the duration of anagen due to accumulated cell, mitochondrial, and ECM damage. Chronological aging also changes the structure of the dermis and the size and position of the follicles [44]. It can also be speculated that the metabolic effects of chronic aging seen in the epidermis will apply to hair bulb matrix cells, which have high energy requirements. Dermal papilla mitochondrial changes are also described as associated with androgenetic alopecia (AGA) [45,46,47]. Hormonal changes are associated with hair loss via influence on metabolic processes such as the thyroid hormone that regulates mitochondrial function and the stress hormone cortisol [48,49].

Hair aging is also associated with stem cell depletion, slower or incomplete DNA, and protein repair processes that result in chronic inflammation with cells having a senescence-associated secretory phenotype (SASP). Structural changes in the scalp and fibrosis in the dermal compartment are seen with aging, and SASP could result in both immune senescence, which could manifest as immune privilege collapse [50,51], and endothelial cell senescence which may restrict the supply of essential nutrients to the follicle [52]. Senescent cells, which no longer divide but remain metabolically active, continue to secrete inflammatory ‘danger’ molecules, and this has spurred the search for senolytic—compounds such as quercitin, fisetin, and dasatinib that can selectively protect cells by either upregulating anti-apoptotic pathways, or by triggering senescent cell death, and so slow down tissue aging in a number of different organs [42,53]. 

One of the key aging propagators is reactive oxygen species (ROS). These form because of the use of oxygen during ATP generation in mitochondria without sufficient natural free radical scavengers, as well as through environmental triggers such as UV irradiation, pollution, and microbial dysbiosis. Oxidative stress at cellular and tissue levels leads to micro-inflammation in tissues, including the scalp, which can have many harmful effects. Oxidative damage to mitochondrial DNA can be measured to reflect aging over prolonged periods, with more damage occurring over time, often as deletions in the mitochondrial genome and mitochondrial damage-associated molecular patterns (DAMPs) [41,42].

Thus, cellular metabolic aging is a key driver of tissue dysfunction and age-related diseases, making it a critical focus in regenerative therapeutics and aging research. This applies very much to the hair follicle. Pharmaceutical therapeutics targeting metabolic aging have not yet been commercialized for hair loss; however, recent studies suggest that managing the integrated stress responses in the hair follicle might help support hair growth [54]. 

### 3.3. The Case for a Multifactorial ‘Hair Longevity’ Strategy

As mentioned, the (approx.) 100,000 follicles on the scalp represent complex regenerative biology, biochemistry, nutrition, metabolism, and recently—the microbiome. Hair longevity represents multiple factors such as hair cycle dynamics, the quality of the hair shaft, how long the hair can be grown, hair color, scalp health, and knowledge on how to minimize triggers for excess shedding due to lifestyle, age, or other factors. 

Therapeutic drug developments for hair loss have focused on single drugs such as 5-αreductase inhibitors to slow early onset pattern hair loss and hair growth stimulants such as minoxidil that reduce the duration of telogen and increase anagen density [55,56,57]. However, these single-drug-target strategies do not work for everyone and require long-term use for continued benefit. Emerging therapeutics such as stem cells [58] and exosomes are still in trial stages [59,60], and biophysical devices are relatively few and have been reviewed elsewhere [29]. 

This has left a gap in the solutions accessible to patients and consumers in which the cosmetics and nutraceuticals markets continue to innovate, driven by the discovery of active ingredients that are introduced in multi-component formulations or by nutrient combining for health and wellbeing benefits. Most hair loss will have a clinical diagnosis and explanation, and the expert clinician will seek to address underlying disease and lifestyle factors (summarized in Table 1). However, with a lack of general access to this clinically led personalized approach, the consumer market for hair thinning and pattern hair loss products has seen the launch of many cosmetic and nutritional multi-active products, often with a combination of phytochemicals, vitamins, minerals, oils and peptides which each claim to address many different pathways in hair growth and tissue maintenance. Several such products have undergone clinical testing and shown some efficacy, and these have been extensively reviewed in the literature [61,62,63,64,65,66,67,68].

The key complementary therapy classes and materials are detailed in Table 2. Phytochemicals, peptides, amino acids, vitamins, and minerals are typically formulated into topical or oral supplement-style products, and cell-derived materials are microinjected. Recent data emerging through the COVID-19 pandemic revealed hair loss associated with lower levels of vitamin D [77,86], and there is considerable evidence that low iron status in women is linked to hair shedding [10,11,72,87]. Zinc and biotin remain popular, but the scientific rationale for their use is weak [88,89,90,91]. 

Products that care for the scalp have grown in number, and scalp health has moved from purely anti-dandruff to the ‘skinification’ of hair, where skincare research and skincare actives are used in scalp/hair care. The composition and resilience of the scalp stratum corneum barrier have been shown to be important in preventing scaling and itching and are linked to better hair growth [103,113,114]. The relevance of the scalp and hair follicle microbiome (and possibly the gut microbiome) to healthy scalp and hair growth is emerging through microbiome sampling studies and the impact of product interventions [115,116,117,118,119]. Sebum synthesis and secretion are impacted physiologically, e.g., through androgens and in older women, where menopause causes sebaceous atrophy and hair that is more ‘dry’ and less manageable. Changes in sebum also impact the microbiome and healthy scalp. Treatments that restore sebaceous activity are a potential route to hair longevity, and there is some evidence to link beauty attributes to sebum levels in women [120,121,122]. Thus, the health of the scalp skin is also important to hair longevity. 

### 3.4. Challenges in Generating and Communicating Clinical Proof of Efficacy 

In the case of multifactorial approaches to hair longevity the rationale for inclusion of components of the formulation, whether topic or oral, are selected to target multiple facets of hair follicle regeneration such as Wnt signaling [123,124]; suspected micronutrient deficiencies such as iron, zinc, and various vitamins (Table 1); building blocks for hair and the extracellular matrix of the dermis such as keratin [104] and collagen [86] and other bioactivities designed to provide hormonal, antioxidant, stress relieving and/or anti-inflammatory activities (Table 2). The design of the efficacy studies ideally should satisfy clinical best practices with a well-defined and balanced starting study population, sufficiently powered for statistical significance; intention to treat placebo-controlled design with quantitatively measurable primary and secondary endpoints and sufficient duration for efficacy to be detected—typically referred to a randomized controlled trial (RCT). The study researchers should also register the trial on a relevant clinical trial register, e.g., the EU Clinical Trails Register (Clinical Trials in the European Union—EMA), and intend to publish the results irrespective of outcomes using the CONSORT framework [125]. However, such studies can be costly; they require experts in hair loss management, such as within a clinical trial center, and require ethical principles as well as ways to ensure participant compliance. Access to technical measurement equipment such as controlled photography, phototrichogram, shed hair collection, and well-designed questionnaires are vital for strong claims, as recently reviewed [126]. The clinical research can either include a proof-of-concept formulation or a final finished product, which will be supplied to patients/participants. Proof-of-concept formulations provide evidence of efficacy for the tested complex, which may then be added to a finished formulation that is not itself then clinically tested before marketing. This approach is often used by specialized ingredient suppliers in the consumer products supply chain. When cost barriers exist, shorter, smaller, or single-leg (non-placebo-controlled) studies are carried out, and whilst less powerful than RCTs, can generate useful data for claims or support the continued development of a product through a future RCT. 

Communicating the benefits of products requires evidence of ingredient(s) and/or finished product efficacy and clarity on who (patient/consumer) would benefit, how long results might take to emerge, and what to look for by way of improvement. RCT data are used by product manufacturers to provide the correct target user with the information they need to make an informed choice, instructions on use and necessary compliance, and to manage expectations on results. For pattern hair loss and hair thinning, results will be seen typically between 4 and 12 months of use. Where hair shedding is measured [103,127], the lessening of this worrying symptom over 3 to 4 months might be the earliest sign of product efficacy. For telogen effluvium, where synchronized exit from the hair cycle has been triggered, the appearance of new short, fine, tipped hairs at the temple and crown are also useful early indicators of restored normal hair growth. 

Thus, the term hair longevity stands for many facets of healthy hair aging, including both physical and emotional aspects. Clinical researchers might also be interested in investigating the emotional benefits of a treatment protocol using standardized instruments for measuring psychological wellbeing, as reviewed by Thadanipon et al., 2021 [128].

## 4. Summary

**Figure 4:**
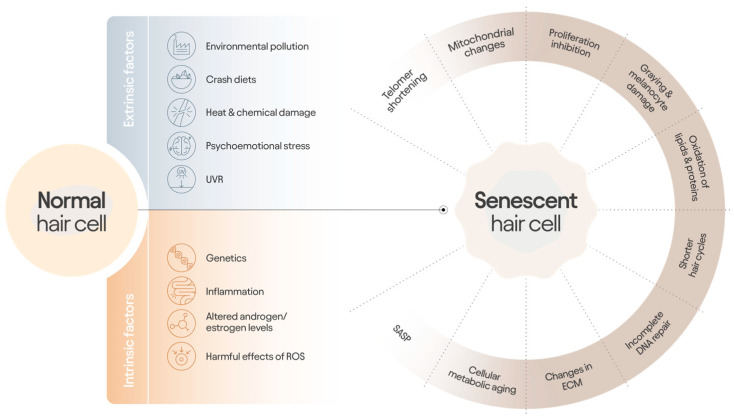
The multifactorial process of hair aging. Intrinsic and extrinsic factors initiate the complex process of hair cell senescence, leading to a great variety of changes.

In this review, we have discussed the evidence for a multifactorial holistic approach to managing hair aging changes, recognizing that hair aging is a complex process encompassing elements of hair cycle alterations, follicle changes, hair fiber changes, external and chronological ‘aging’ factors affecting cell physiology and metabolism, as summarized in Figure 4. Hair aging also includes an emotional component, where the emotional response to hair thinning, balding, patchy hair loss, shedding, and loss of hair volume are important for a person’s self-esteem and psychological wellbeing. Whilst many products are being developed to address pattern hair loss and hair thinning, not all are tested through RCTs, and often, claims are based on limited data linked to in vitro testing of ingredient(s). Thus, when there is ready access to information on the internet without clarity on its veracity or quality, there is misinformation. However, there is an opportunity to combine expert diagnosis—Dermatologist- or Trichologist-led—with quality data provided through peer review publications of RCTs—with artificial intelligence—to educate and guide the consumer through choices to help each person achieve healthy hair longevity. 

## Figures and Tables

**Table 1:** The main co-morbidity factors that may have hair loss as a presenting symptom.

Co-Morbid Disorders with Alopecia	Symptom	References
Thyroid dysfunction (hyper/hypo)	Excess hair shedding	[69]
Low iron status (Ferritin < 70 ng/mL)	Hair thinning	[9,10,11,69,70,71,72,73]
Hormonal disturbances, e.g., Menopause and PCOS	Hair thinning/pattern hair loss, hirsutism	[6,65,74,75]
Vitamin D deficiency	Hair thinning	[76,77,78,79,80]
Genetic predisposition	Pattern hair loss	[81,82]
Age	Senile hair changes	[37,83,84]
Stress—psychoemotional	Excess hair shedding	[85]

**Table 2:** The key complementary therapy classes and materials. Specific ingredients are used within complex formulations. Daily dose information provided where available. No trade names disclosed.

Complimentary Therapy Classes and Ingredients
Cell-derived; Stem and DP cell stimulants Exosomes (foreskin-derived mesenchymal stromal cells). Exosome preparation and characterization described by Ersan et al. [59], and reviewed by Shah [60] Platelet-rich plasma (PRP) prepared from whole blood as described by Pakhomova, et al. [92] Adipose stem cells (follicle-associated and dermal white adipose, prepared as described by Andjelkov [93]) Injectable platelet-rich fibrin (i-PRF) was prepared as described by Lu, et al. [94]
Marine based extracts Marine hydrolyzed collagen (300 mg/dose) included in a one-a-day supplement containing taurine, cysteine, methionine, iron, and selenium (Milani, et al. [87]) Marine protein complex, shark and oyster extract were tested by Augustyniak [95] in vitro on dermal papilla cells at a dose reflective of the commercial in vivo dose (600–950 mg/dose)
Phytochemicals Caffeine used in ex vivo culture at 0.0005–0.001% and 1–2.5% in vivo in leave-on and rinse-off, as summarized by Daniels, et al. [61] Phytoestrogens/isoflavones. Equol 7-hydroxy-3[40hydrozyphenyl]-chroman used within a proprietary supplement by Brotzu, et al. [96] EGCG—1 μM ex vivo and 10% in vivo in studies as reviewed by Daniels, et al. [61] *Silybum marianum* extract (SME), manganese PCA (MnPCA), and a *Lespedeza capitata* extract (LCE) dosed in vitro at 30 μg/mL Bacqueville [97])
Anti-androgens Saw Palmetto (*Serenoa repens*) has been used in various formulations, e.g., see Sudeep, et al. [98], Prager, et al. [99] and Narda, et al. [100] Available at 320mg/dose Pumpkin seed oil (*Cucurbita pepo*) is also associated with treatments for hair loss, available at 300 md/dose; see Ibrahim, et al. [101]
Anti-inflammatories Flavonoids use is reviewed by Bassino, et al. (e.g., Quercitrin (quercetin-3-O-rhamnoside)) Herbal extracts and mixtures, e.g., as described by Bhatia, et al. [67], Nam. et al. [102], Feldman, et al. [62,63]
Antioxidants Many materials act as antioxidants Davis, et al. tested a mixture containing piroctone olamine, zinc pyrithione, zinc carbonate, niacinamide, panthenol and caffeine [103] A supplement (Ablon, et al. [65]) contains anti-inflammatory, adaptogenic (anti-stress), antioxidant, and dihydrotestosterone-inhibiting ingredients
Peptides and amino acids Marine-derived collagen has been used in formulations as described by Milani [87] and Augustyniak [95] A natural keratin hydrolysate supplement was tested in women by Tursi, et al. [104] Pentapeptide (Gly-Pro-Ile-Gly-Ser) was effective topically, as shown by Iwabuchi, et al. [105] Peasprout (100 mg) given orally daily for 8 weeks [106]
Vitamins and minerals Vitamin D deficiency has been associated with hair shedding, and Sattar, et al. [77] showed a benefit of 6 oral vitamin D(3) (200,000 IU) doses given every 2 weeks in women improved hair shedding Biotin, and sometimes B12, are included in hair loss treatments, but evidence is lacking, as shown by Abdel, et al. [91] and reviewed by Almohanna, et al. [107]. Zinc deficiency has been reported with telogen effluvium [88] and Durusu, et al. [69], zinc supplements have been reported to lessen hair loss [89], and low iron/ferritin levels are considered by some as a risk for hair loss [70,71,73]. Though included in hair loss treatments, less evidence is available for selenium, manganese, and copper
Small molecules Adenosine has been clinically tested at 0.75% by Oura, et al. [108] and Kim, et al. [109] and 0.2% with caffeine by Chen, et al. [110] Spermidine supplementation via an oral supplement was shown by Rinaldi, et al. [111] to be beneficial for hair loss, and N(1)-methylspermidine prolongs hair follicle maintenance ex vivo [112]

## Data Availability

The data generated and analyzed during this study are included in this published article.

## Abbreviations

The following abbreviations are used in this manuscript:

5αR	5-α reductase
AGA	Androgenetic alopecia
DAMPs	Damage-associated molecular patterns
DHT	Dihydrotestosterone
DP	Dermal papilla
ECM	Extracellular matrix
FPHL	Female pattern hair loss
MPHL	Male pattern hair loss
PRP	Platelet-rich plasma
RCT	Randomized control trial
ROS	Reactive oxygen species
SASP	Senescence-associated secretory phenotype
UVR	Ultraviolet radiation
